# Velpeau on the Treatment of Erysipelas

**Published:** 1846-12

**Authors:** 


VELPEAU *on the treatment of Erysipelas.—From report of cases treated by* VELPEAU *in the Hospital La Charite, Paris, by correspondent of the Boston Med. and Surg. Journal.*

"In respect to treatment, erysipelas presents this remarkable peculiarity, that it is not arrested by any kind of treatment whatever. We have tried every imaginable topical application without having arrested its advance. The mercurial ointment, lard, ointment with calomel, sulphate of copper, diluted acids, the nitrate of silver, of which much has been said, and upon which an American physician has written a whole volume; blisters, sinapisms, &c., all these means have failed. One unguent alone appears to us to have any effect upon erysipelas; it is the ointment of the sulphate of iron. ℞. Sulph. ferri, eight to ten grains; axunge, thirty to forty grains. A solution of ten grains to one hundred grains of water may be applied to the skin by means of moistened compresses. This salt evidently has an influence upon erysipelas; the teguments with which it comes in contact become pale and shrivelled, but it has not more than the remedies above mentioned the property of limiting the disease. This application has not then in reality any great importance; we see in some patients the general symptoms persist, and even increase in spite of the disappearance of the redness, which proves that it is a general disposition which produces it, and which in fact alone renders it dangerous. Nevertheless, we employ the sulphate of iron in erysipelas, as it renders more service, but what is required is a general, *constitutional* remedy. It appears singular that of the five patients who entered the hospital for erysipelas, none died, while eight of the fifteen who were attacked with the same affection, being already in the wards, have died. This appears enormous, but it is very easily explained. The five patients who entered for erysipelas had no other disease; but although it may sometimes terminate seriously, recovery is much more usual. Of the fifteen patients who were attacked with erysipelas in the hospital, not one was affected with this phegmasia alone; some had undergone operations, one had a peritonitis, another a pneumonia, others suppuration. That which caused these patients to die was not more the erysipelas than the other diseases which they presented. Thus it may be established, as a precept, in surgery and medicine, that erysipelas as a complication is a very serious disease and destroys a great number of patients, if not by itself, at least by reason of its developing itself in the midst of circumstances already grave."

